# On the morphology of electrostatic solitary waves in the Earth’s aurora

**DOI:** 10.1038/s41598-022-23095-y

**Published:** 2022-10-28

**Authors:** Steffy Sara Varghese, Kuldeep Singh, Ioannis Kourakis

**Affiliations:** 1 Space and Planetary Science Center, Khalifa University of Science and Technology, P.O. Box 127788, Abu Dhabi, United Arab Emirates; 2 Department of Mathematics, Khalifa University of Science and Technology, P.O. Box 127788, Abu Dhabi, United Arab Emirates

**Keywords:** Plasma physics, Space physics

## Abstract

Electrostatic solitary waves (ESWs) have been detected in abundance in Space plasma observations, both by satellites in near-Earth plasma environments as well as by planetary missions, e.g. Cassini in Saturn or MAVEN in Mars. In their usual form, these are manifested as a bipolar electric field corresponding to a bell-shaped pulse in the electrostatic potential. Recent studies have suggested the existence of alternative forms of ESWs, including flat-top solitary waves (FTSWs) and supersolitary waves (SSWs), both of which are often encountered in Space observations such as in polar cap boundary layer, the auroral acceleration region and elsewhere. This article focuses on the existence and characterization of different types of electrostatic solitary waves in multicomponent Space plasmas. Relying on a multi-fluid plasma model, comprising two types of ions and two different electron populations, we have identified the conditions for existence of flat-top solitary waves and supersolitons, in contrast to “standard" solitary waves. Both ion species are models as cold fluids, for simplicity. Our analysis reveals that the coexistence of the two electron populations is pivotal for the formation of such non-standard electrostatic structures, and that their characteristic parameters (temperature, density ratio) plays a decisive role in their generation and structural characteristics. Nonetheless, while supersolitary waves may exist in a wide range of parameter values (as confirmed by earlier theoretical studies), it appears that flat-top solitary waves will occur in a narrow window in the parameter region, which may explain their scarce (but non-negligible) frequency of observation. Our theoretical findings confirm and validate the existence of alternative (non-conventional) ESW waveforms in auroral plasma (in addition to the ubiquitous bipolar electric field form), where such an electron coexistence is typically observed.

## Introduction

Electrostatic Solitary Waves (ESWs) are an ubiquitous occurrence in the Earth’s magnetosphere, and analytical methodology adapted from coherent nonlinear localized structures have proven invaluable in their interpretation^1–4^. These are often interpreted as solitons, i.e. exact solutions of integrable nonlinear partial differential equations (PDEs) that are characterized by remarkable stability properties, in that they sustain their profile against mutual interactions and external perturbations^5^. Since an exact mathematical description of such localized waveforms cannot be developed, given the complexity of physical mechanisms in play, these are usually referred to as solitary waves. (The two terms may be interchangeably used in this paper, even though we refer to structures whose integrability properties have not been established, and are therefore not necessarily solitons, in the strict sense). ESWs are associated with localized coherent structures in the electric (E-) field data recorded by the satellites in the Earth’s magnetosphere. Once integrated, these E-field excitations are shown to be related to localized waveforms (typically, pulses) in the electrostatic (ES) potential, in turn associated with (co-propagating) localized density disturbances.

Although weak amplitude excitations are adequately modeled by nonlinear PDEs^6^, and those prediction have been corroborated by experimental studies^7^, the obtained solutions have their physical limitations; in particular, those theories predict only structures propagating slightly above the plasma sound speed, thus failing to account for larger amplitude super-acoustic solitary waves. The latter are modeled by the (so called Sagdeev-type) pseudopotential technique^8^, which not only succeeds in predicting the stationary profile of the plasma variables (localized disturbances), but also enables a study of the existence conditions for such structures, from first principles^9,10^.

Less than a decade ago, the pseudopotential technique for large-amplitude electrostatic solitary waves received new impetus, thanks to the prediction of non-conventional waveforms (e.g. super-solitary waves, also known as "supersolitons") that has reignited interest in the subject. The supersoliton concept was proposed by Dubinov and Kolotkov^11^, while studying solitary waves in multicomponent plasma configurations. They found that, under certain conditions, the “Sagdeev" pseudopotential form can support three consecutive local extrema, causing wiggles or knee-like structures (bumps) superposed on the (usually smooth, monotonic on each side) potential profiles of the corresponding solutions (obtained numerically). This results in the creation of additional extrema in the bipolar electric field forms. Shortly after the first prediction of supersolitons in a five species plasma, Verheest et al.^12^ showed that such forms also occur in plasmas with as few as three species. In the years that followed, various theoretical and numerical studies were carried out to understand the fundamental properties of super-solitary waves in different plasma systems^12–30^ and, interestingly, these were eventually detected in numerical experiments^31^.

Although the concept of FTSW is by now established in the field of non-linear optics and photonics^32,33^, it has been less explored in plasma physics. Interestingly, FTSW have been obtained as a special solution of the extended Korteweg–de Vries (eKdV) (or Gardner) equation^34^. Various kinds of extra-nonlinear localized forms were discussed by Steffy and Ghosh^35^, who emphasized how their morphology differs from conventional solitary wave and double layer structures. One such non-conventional nonlinear structure, the flat-top solitary wave (FTSW), is characterized by a square (table-top like) shaped potential profile. Interestingly, this possibility had been discussed in passing in an earlier series of papers by Hellberg et al and by Verheest et al, where the boundary separating supersolitons from regular solitary waves was shown to be associated with a triple root of the pseudopotential; see e.g. Ref.^21^ (Figures 2 and 3 therein),^18^ (see Figure 7 therein) or^12^ (see Fig. 4 therein). More recently, Verheest et al.^36^, investigated the possibility for the existence of FTSW in a multi-component plasma model consisting of hot electrons, nonthermal (Cairns-distributed) positive ions, and cold, negatively charged dust grains. In their analysis they have reported FTSW structures as dependent on parameter values in a very sensitive way, casting some doubt on whether they may be observed at all (given the subtle dependence on the plasma background properties).

In the Earth’s auroral zone, apart from the admixing of hot magnetospheric electrons with a cold component originating from the ionosphere, plasma has been found to have a significant contribution of oxygen (O$^{+}$) ions along with its usual proton (H$^{+}$) population. There have been various satellite observations of electrostatic solitary waves in this region^37–40^. It is interesting to consider how these solitary wave pulses function in contributing to energy transfer and exchange between particles, as well as how they promote anomalous resistivity. Apart from conventional bipolar pulses, satellite observations have detected the presence of stretched and asymmetric bipolar pulses within this region^4^. For a stretched bipolar pulse, the distance between the successive peaks is relatively large compared to the characteristic width of each peak. Because of their "stretched" look, such bipolar pulses are often termed as "stretched bipolar" or "dispersed bipolar", or "offset bipolar pulses" (ofbp) in the literature. Inspired by these observations, we have developed a two-fluid model to investigate the occurrence of flat-top solitons and supersolitary waves in auroral plasma, from first principles. Inspired by the coexistence of ions that has been observed e.g. in the auroral acceleration region^37^, we have adopted a two-fluid model. For simplicity in the analysis, both ions will be modeled as cold fluids (an assumption to be relaxed in future work). A coexistence of two electron populations at thermal equilibrium (say, ‘cold’ and ‘hot’ electrons) is incorporated in the model. We are interested at this stage in exploring the very basic mechanism(s) contributing to the formation of such “exotic" solitary wave structures. An investigation of the conditions for the existence of such structures will reveal that the coexistence of the two electron populations is a crucial requirement for the formation of these structures, while their characteristics (relative temperature and concentration ratios) play a deterministic role in the generation of flat-top solitary waves (FTSWs).

## The model

We have considered an infinite, homogeneous, collisionless, unmagnetized plasma comprising of two types of (positively charged) ions, in addition to two electron populations at thermal equilibrium (but with different temperature). Both of the electron components obey a Maxwell-Boltzmann distribution, while the ions are modeled as cold fluids. The ion fluid equations read

1a $$\begin{aligned}&\frac{\partial n_{ij}'}{\partial t'}+\frac{\partial (n_{ij}' u_{ij}')}{\partial x'}=0 \, , \end{aligned}$$

1b $$\begin{aligned}{}&\frac{\partial u_{ij}'}{\partial t'}+u_{ij}'\frac{\partial u_{ij}'}{\partial x'}=-\frac{z_{ij}e}{m_{ij}}\frac{\partial \phi '}{\partial x'}, \end{aligned}$$

where the subscript $'i'$) is for ‘ions’, i.e. $'ij'$ denotes either the lighter ions (j = 1) or the heavier ion component (j = 2). (Certainly, this mass distinction is arbitrary, and will play no role, really, as expected. Let us just keep in mind that ions 1 are e.g. hydrogen ions, while ions 2 may be either helium or oxygen ions, for instance.) Accordingly, $n_{ij}$ denotes the number density of the corresponding (*j*-th) ion species, $u_{ij}$ the corresponding fluid speed, $z_{ij}$ the charge multiplicity (state) of ion species 1 or 2, *e* the elementary (electron) charge, and $\phi$ is the electrostatic potential. In the above equation, the prime ($'$) represents the physical state variables, to be distinguished from their normalized counterparts (where primes will be dropped) later, upon rescaling by suitable physical quantities (scales) The electron density for the two electron populations, distinguished by the subscripts ‘c’ (for cold) and ‘h’ (for hot) are:

2a $$\begin{aligned}{}&n'_{ec}=n_{ec0}\, \exp \left( \frac{e\phi '}{K_B T_{ec}}\right) , \end{aligned}$$

2b $$\begin{aligned}{}&n'_{eh}=n_{eh0}\, \exp \left( \frac{e\phi '}{K_B T_{eh}}\right) \end{aligned}$$

where $n_{ec0}$ and $n_{eh0}$ denote the corresponding equilibrium densities, and $T_{ec}$ and $T_{eh}$ are their corresponding temperature(s) ($K_B$ obviously denotes Boltzmann’s constant). The system of equations is closed by Poisson’s equation

3 $$\begin{aligned} \frac{\partial ^2 \phi '}{\partial x^2}=-\frac{e}{\varepsilon _0}\left[ z_{i1}n_{i1}'+z_{i2}n_{i2}'-(n_{ec}'+n_{eh}')\right] . \end{aligned}$$

For the sake of analytical convenience, we shall now normalize Eqs. (1)–(3) by scaling over appropriate plasma quantities. The ion number densities $n'_{ij}$ are scaled by the corresponding equilibrium densities ($n_{ij0}$, respectively, for $j=1, 2$). The electron densities $n'_{ec}$ and $n'_{eh}$ are scaled by the (total) equilibrium electron density $(n_{e0}) = n_{ec0}+n_{eh0}$. The ion fluid speed(s) $u'_{i, j}$ will be scaled by the characteristic speed (scale) $c_{i1}=\sqrt{\frac{z_{i1} K_B T_*}{m_{i1}}}$. Time $(t')$ will be scaled by the (light ion) plasma frequency $\omega _{pi1}=\sqrt{\frac{n_{i10}(z_{i1}e)^2}{\varepsilon _0 m_{i1}}}$. Length (x’) will be scaled by the characteristic length $\lambda _*=\sqrt{\frac{\varepsilon _0 K_B T_*}{n_{i10}z_{i1}e^2}}$. Finally, the ES potential $(\phi ')$ will be scaled by $\frac{K_B T_*}{e}$. It is obvious that we have chosen the first type of ions (the “light" ions) as point of reference, i.e. choosing to scale time by the (ion-1) plasma period (inverse plasma frequency), length by what would be the Debye screening length (in the absence of ions-2) and, finally, the fluid speed variables by what would be (but is not, in our case) the sound speed, in the absence of the second (“heavy") ion population. In other words, upon switching off the second ion fluid (i.e. setting $n_{i20} = 0$), one readily recovers a classical textbook electron-ion plasma and its well known properties.

Imposing the above normalizations, we obtain a set of dimensionless fluid equations for the ion species in the form

4a $$\begin{aligned}{}&\frac{\partial n_{i1}}{\partial t}+\frac{\partial (n_{i1}u_{i1})}{\partial x}=0, \end{aligned}$$

4b $$\begin{aligned}{}&\frac{\partial u_{i1}}{\partial t}+u_{i1}\frac{\partial u_{i1}}{\partial x}=-\frac{\partial \Phi }{\partial x}, \end{aligned}$$

4c $$\begin{aligned}{}&\frac{\partial n_{i2}}{\partial t}+\frac{\partial (n_{i2}u_{i2})}{\partial x}=0 , \end{aligned}$$

4d $$\begin{aligned}{}&\frac{\partial u_{i2}}{\partial t}+u_{i2}\frac{\partial u_{i2}}{\partial x}=-\frac{Q}{\mu }\frac{\partial \Phi }{\partial x}, \end{aligned}$$

where we have defined the charge and mass ratios:

$$\begin{aligned} Q = \frac{z_{i2}}{z_{i1}} ; \qquad \mu =\frac{m_{i2}}{m_{i1}}. \end{aligned}$$

Similarly the normalized electron densities are written in a dimensionless form as

5a $$\begin{aligned} n_{ec}=&\zeta \, \exp \left( \frac{T_*}{T_{ec}}\Phi \right) , \end{aligned}$$

5b $$\begin{aligned} n_{eh}=&\nu \, \exp \left( \frac{T_*}{T_{eh}}\Phi \right) , \end{aligned}$$

where

$$\begin{aligned} \zeta = \frac{n_{ec0}}{n_{e0}} ; \qquad \nu =\frac{n_{eh0}}{n_{e0}} = 1 - \zeta ;\qquad T_*=\frac{1}{1+ Q \delta } T_{eff}=\frac{1}{1 + Q\delta } \left( \frac{\zeta }{T_{ec}}+\frac{\nu }{T_{eh}}\right) ; \qquad \delta =\frac{n_{i20}}{n_{i10}}. \end{aligned}$$

Finally, the (normalized) form of Poisson’s equation reads

6 $$\begin{aligned} \frac{\partial ^2 \Phi }{\partial x^2}=-\left[ n_{i1}+Q\delta n_{i2}-(1+Q\delta )\left\{ \zeta exp\left(\frac{T_*}{T_{e,c}}\Phi\right) +\nu exp \left(\frac{T_*}{T_{e,h}}\Phi\right) \right\} \right] . \end{aligned}$$

## Linear dispersion relation

Let us recover dimensions for a moment. By linearizing the original fluid equations Eqs. (1)–(3) and assuming harmonic excitations $\sim \exp [i (k x - \omega t)]$ for all state variables, we obtain a linear dispersion relation in the form

7 $$\begin{aligned} \omega ^2=\omega _{peff}^2 \, \frac{k^2}{k^2+\lambda _{D eff}^{-2}} \end{aligned}$$

where $\omega _{p eff}^2=\omega _{pi1}^2+\omega _{pi2}^2$ and $K_{D eff}^{-1} = \lambda _{D eff} = \left( \frac{e^2}{\varepsilon _0}\left( \frac{n_{ec0}}{K_B T_{ec}}+\frac{n_{eh0}}{K_B T_{eh}}\right) \right) ^{-1/2}$ is the effective (Debye) screening length in our plasma model. From Eq. (7), the acoustic speed in our multicomponent plasma model ($C_s$) can be obtained as

8 $$\begin{aligned} \lim _{k \rightarrow 0} \left( \frac{\omega }{k}\right) = C_s = \left( \frac{\omega _{pi1}^2+\omega _{pi2}^2}{K^2_{Deff}}\right) ^{1/2} =\left( \frac{1+\delta Q^2/\mu }{1+Q\delta }\right) ^{1/2}\left( \frac{z_{i1}K_B T_{eff}}{m_{i1}}\right) ^{1/2} . \end{aligned}$$

In Eq. (8), if one sets $\delta =0$ (single ion limit), then

9 $$\begin{aligned} C_{s}=\left( \frac{z_{i1}K_B T_{eff}}{m_{i1}}\right) ^{1/2}, \end{aligned}$$

which corresponds to the acoustic speed of a two electron temperature-single ion plasma system^41,42^. If, furthermore, one sets $\zeta =0$ (thus eliminating the cold electron component), then Eq. (8) becomes

10 $$\begin{aligned} C_{s} =\left( \frac{z_{i1}K_B T_{eh}}{m_{i1}}\right) ^{1/2}, \end{aligned}$$

thus recovering the known expression for the acoustic (sound) speed in electron-ion plasma.

## Nonlinear analysis

Anticipating stationary profile solutions, the fluid equations will be expressed in a reference frame by applying the transformation from $\{x, t\}$ to $\eta =x - V t$, where *V* is the velocity of the solitary wave (i.e. the pulse speed $v_{pulse}$, scaled by $c_{i1}=\sqrt{\frac{z_{i,1} K_B T_*}{m_{i,1}}}$, as shown above). Note, for the sake of rigor, that the variable *V* is not the “Mach" number, as often (erroneously) stated in various works. Actually, the true Mach number in our case would be $M = v_{pulse}/C_s = V \, \left( \frac{1+\delta Q^2/\mu }{1+Q\delta }\right) ^{- 1/2}$.

By adopting vanishing boundary conditions for the density and fluid speed disturbance, viz. $u_{ij} \rightarrow 0$, $n_{ij} \rightarrow 1$ and $\Phi \rightarrow 0$ as $|x| \rightarrow \infty$, one finds the perturbed densities for the two ion species ($j =1, 2$) as

11a $$\begin{aligned} n_{i1}&=\left( 1-\frac{2\Phi }{V^2}\right) ^{-1/2} \end{aligned}$$

11b $$\begin{aligned} n_{i2}&=\delta \left( 1-\frac{2\Phi Q}{\mu V^2}\right) ^{-1/2} \end{aligned}$$

and the corresponding fluid speed(s) as,

12a $$\begin{aligned} u_{i1}&=V\left[ 1-\left( 1-\frac{2\Phi }{V^2}\right) ^{1/2}\right] , \end{aligned}$$

12b $$\begin{aligned} u_{i2}&=V\left[ 1-\left( 1-\frac{2Q\Phi }{\mu V^2}\right) ^{1/2}\right] . \end{aligned}$$

Substituting with the definition of the characteristic temperature $T_*$ in Eq. (5), the number densities of the cold and hot electron populations can be rewritten as

13a $$\begin{aligned} n_{ec}&=\zeta \, \exp \left( \frac{\Phi }{(1+Q\delta )(\zeta +\nu \beta )}\right) , \end{aligned}$$

13b $$\begin{aligned} n_{eh}&=\nu \, \exp \left( \frac{\Phi \beta }{(1+Q\delta )(\zeta +\nu \beta )}\right) , \end{aligned}$$

where  $\beta ={T_{ec}}/{T_{eh}}$ is the cold-to-hot electron temperature ratio.

Substituting into Poisson’s equation now leads to

14 $$\begin{aligned} \frac{\partial ^2 \Phi }{\partial \eta ^2} = n_e - n_i = -\frac{\partial S(\Phi )}{\partial \Phi }, \end{aligned}$$

which, upon integration, leads to the pseudo-energy balance relation

15 $$\begin{aligned} \frac{1}{2}\left( \frac{d\Phi }{d\eta }\right) ^2+S(\Phi , V)=0. \end{aligned}$$

The pseudopotential function $S(\Phi , V)$, obtained upon integrating Poisson’s equation (14), reads

16 $$\begin{aligned} S(\Phi , V)= {} -\Big (1+Q\delta \Big )^2 \Big (\zeta +\nu \beta \Big)& \Bigg [\zeta \Big \{\exp \left(\frac{\Phi }{(1+Q\delta )(\zeta +\nu \beta )}\right)-1\Big \} +\frac{\nu }{\beta }\Big \{\exp \left(\frac{\beta \Phi }{(1+Q\delta )(\zeta +\nu \beta )}\right)-1\Big \}\Bigg ] \nonumber \\& + V^2 \Bigg [1-\Big (1-\frac{2\Phi }{V^2}\Big )^{1/2}\Bigg ]+ \delta V^2 \mu \Bigg [1-\Big (1-\frac{2Q\Phi }{\mu V^2}\Big )^{1/2}\Bigg ] \, . \end{aligned}$$

Note that the pseudopotential $S(\Phi , V)$ is a function of the (dimensionless) electrostatic potential $\Phi$ and of the pulse speed *V*, for a given plasma configuration (i.e. for fixed values of all other parameters). It is straightforward to distinguish the contribution from the cold and the hot electrons (first and second term/s, within square brackets), followed by the first (light) and the second (heavy) ion component.

## Conditions for existence of localized modes

In order to obtain the existence of a solitary wave solution, to be obtained upon numerical integration of either the pseudo-equation of motion (Eq. 14), or the energy balance Eq. (15), the potential form $S(\Phi )$ (Eq. 16) must satisfy the following requirements:

17 $$\begin{aligned} S(\Phi =0, V) =\frac{\partial S}{\partial \Phi }\Big |_{\Phi =0} = 0 \, , \qquad \frac{\partial ^2 S(\Phi , V)}{\partial \Phi ^2}\Big |_{\Phi =0} < 0 \, \qquad \forall \, V \in \mathfrak {R}, \end{aligned}$$

ensuring that a local maximum exists at the origin ($\Phi = S(\Phi , V) = 0$), that represents equilibrium. Furthermore, a (non-zero) root is assumed to exist, i.e. $( S(\Phi _0, V)=0 )$ for some value $\Phi = \Phi _0$, where the curve crosses the horizontal axis. Since all variables are real, Eq. (15) implies that $S(\Phi ) < 0$ for $0< \Phi < \Phi _0$, hence the root $\Phi _0$ represents the amplitude of the solitary wave. In other words, the dynamics will "visit" (only) the region between the origin and the first root $\Phi _0$.

Note, for comparison, that in the case of a double layer (DL), i.e. a “kink-soliton", representing a localized transition between two different asymptotic values, there is no returning to the equilibrium state. The latter requirement is then modified as follows:

18 $$\begin{aligned} \frac{\partial S(\Phi _{DL}, V)}{\partial \Phi } = \Delta n_{DL}=0, \end{aligned}$$

where $\Phi _{DL}$ is the DL amplitude and $\Delta n_{DL}$ is the charge separation at the double root $\Phi _{DL}$ (where $S(\Phi _{DL}, V)=0$).

In the case of a flat-top solitary wave (FTSW), the root is a triple root, and the latter requirement becomes

19 $$\begin{aligned} S(\Phi _0, V)=0, \qquad \frac{\partial S}{\partial \Phi }\Big |_{\Phi _0}=\varepsilon ,\qquad \frac{\partial ^2 S}{\partial \Phi ^2}\Big |_{\Phi _0}=\vartheta , \qquad \varepsilon ,\vartheta \ll 1, \end{aligned}$$

where $\varepsilon$ and $\vartheta$ are small positive (real) numbers^35^. In practice, the first and second derivatives of the pseudopotential function $S(\phi , V)$ must vanish (or have a negligible value) at the root (of *S*), identifying this point as a triple root structure^24^.

Supersolitons (or supersolitary waves, SSW) are associated with Sagdeev pseudopotential function forms that possess three consecutive local extrema. Hence, for a SSW to occur, the Sagdeev pseudopotential $S(\phi )$ must have a suitable analytical form, such that $\frac{\partial S}{\partial \Phi }$ and $\frac{\partial ^2 S}{\partial \Phi ^2}$ possess 4 and 3 roots, respectively, between the origin and its main root where it crosses the $\phi$ axis (note that one of these requirements entails the other, since $S(\phi )$ and its derivatives are continuous differentiable functions)^12,13,20,43^.

## Results and discussion

Nonlinear coherent structures, such as solitary waves and Double Layers (DLs), have been analyzed in various studies in the past. For a review of the generic form of the pseudopotential curve and the associated dynamics, the reader is referred to^9^. On the other hand, non-conventional structures such as e,g, FTSWs have not been paid the attention they deserve. In an earlier study, Steffy and Ghosh^35^ suggested a link between FTSWs and supersolitary waves (SSWs) in a multi-species plasma consisting of warm ions and electrons. Verheest et al.^36^ followed by investigating FTSW solutions in a dusty plasma system contains three species hot electrons, Cairns non-thermal positive ions, and cold, negatively charged dust grains. Contrary to those earlier works, where ions were modeled as warm fluids, we want to focus our attention here on establishing the basic plasma requirements for the existence of FTSW. In simple words, we wish to check whether ion temperature in an significant parameter in the occurrence of FTSW in a multi-species plasma.

In the parametric analysis that follows, we are modelling the signatures of bipolar and stretched bipolar parallel electric field pulses observed in the auroral acceleration region a plasma comprising hydrogen ($H^+$) and (singly ionized) oxygen ($O^+$) ions. In the auroral acceleration region the plasma has been found to contain a significant contribution of $O^+$ ions along with its usual proton ($H^+$) population. Moreover, there is an admixing of hot energetic electrons originating from the plasma sheet, cold electrons which are assumed to be produced by the ionospheric back scattering or turbulent scattering of plasma sheet electrons^44^. In this region the Langmuir probe measurements of the S3-3 satellite confirmed^45^ that the ambient density in this region is of the order of $n_0 = 5$–$50 cm^{-3}$. For our analysis we have chosen an electron density $n_0 = 10\;cm^3$, and assumed a very low concentration of cold electron (viz., $0.001\%$ of $n_0$), leading to $\zeta = 0.0001$ in our parametrization. In this region, protons are considered to be the dominant species, while the concentration of $O^+$ will be variable depending on the geomagnetic activity^37^. Hence, in the analysis, we have assumed a small presence of $O^+$ ions ($0.01\%$ of $n_0$) and a dominant concentration of $H^+$ of $99.99\%$, leading to $\delta =0.01$, in our notation. Since both ions are singly charged we shall therefore take the ion charge state(s) to be $z_{i, 1} = z_{i, 2} = 1$ and the mass ratio equal to $\mu = 16$.

### Topology of the pseudopotential curve

In this section, we shall focus on selected combinations of values for the plasma configuration parameters, to illustrate the point(s) we want to make, regarding the existence of pulses of various types and the possible transition between one type and another.

**Figure 1:**
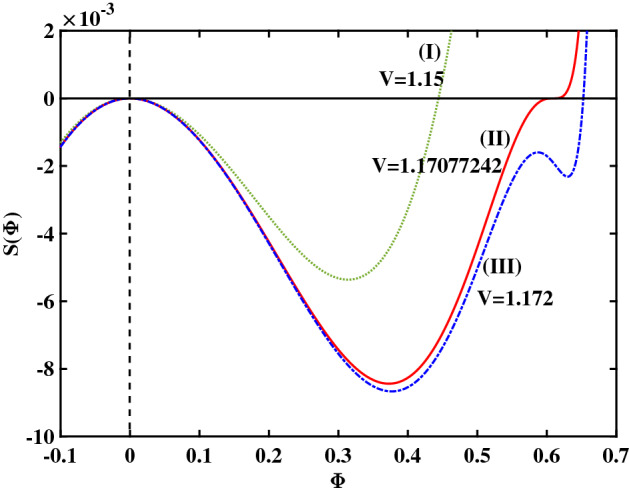
Sagdeev pseudopotential profiles for different V values; the depicted curves represent (I) RSW (in green color) for $V = 1.15$, (II) FTSW (in red color) for $V =1.17077242$, and (III) SSW (in blue color) for $V = 1.172$. The remaining parameter values are: $\beta =0.06439$, $\zeta =0.0001$, $\delta =0.01$, $\mu =16$, and $Q=1$.

To begin with, in Fig. 1 we have plotted a set of solutions supported by the plasma configuration under consideration, for a cold-to-hot electron temperature ratio $\beta =0.06439$. For the entire set of curves shown in that plot (varying the pulse speed *V*), we have kept the remaining plasma parameters constant: cold electron density $\zeta =0.0001$ and (2nd to 1st) ion species density ratio $\delta =0.01$. Recall that the amplitude of the solitary structure (pulse) is given by the root of the pseudopotential $S(\phi )$, which actually increases as *V* increases (so that stronger excitations will be faster too). In Fig. 1, the leftmost curve (I) ($V=1.15$) corresponds to a regular solitary wave (RSW), whereas the middle curve (II) ($V=1.17077242$) represents a flat-top solitary wave (FTSW) and curve III ($V=1.172$) represents a supersolitary wave (SSW). Comparing the respective profiles obtained in the different plots, we note that a FTSW (curve II) is associated with a pseudopotential curve that crosses the horizontal axis almost at “grazing incidence" (i.e. the derivative at the root is almost zero). In the case of a SSW—curve (III)—the Sagdeev pseudopotential posseses two additional extrema (a local maximum and a local minimum) in addition to the expected two extrema featured by all curves. As shown in Fig. 1, even a marginal increment in the pulse speed value beyond $V \simeq 1.171$ (where the flat-top curve shape occurs) leads to a significant change in the topology of the pseudopotential $S(\Phi )$ curve.

**Figure 2:**
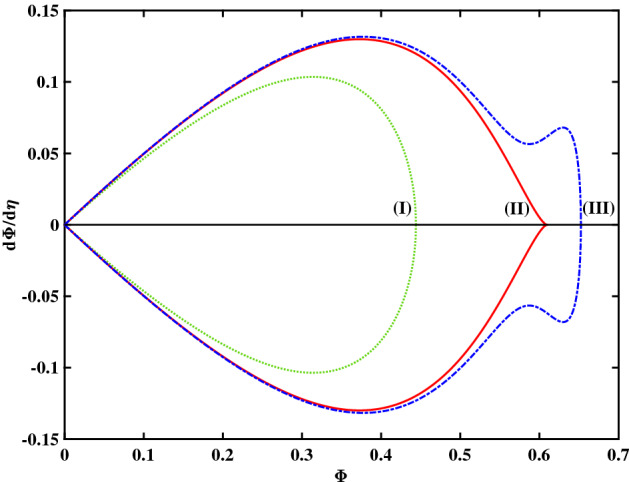
Phase portrait profiles for different V values; the depicted curves represent (I) RSW (in green color), for $V = 1.15$, (II) FTSW (in red color), for $V =1.17077242$, and (III) SSW (in blue color), for $V = 1.172$. The remaining parameter values are: $\beta =0.06439$, $\zeta =0.0001$, $\delta =0.01$, $\mu =16$, and $Q=1$.

In Fig. 2 we have plotted the phase portrait profiles corresponding to the three curves depicted in the first figure. The same notation (curve labels) and color code have been used, for quick reference. We notice the structural difference between the leftmost “conventional" solitary wave profile (curve (I)) and the characteristic wiggly structure associated with supersolitons (SSW, curve (III)). As for the middle curve (II), notice the characteristic way it approaches the horizontal axis, which may be considered as the signature of flat-top solitary waves. It appears that the FTSW condition separates the regions where regular SW occur from the region where SSW may exist.

**Figure 3:**
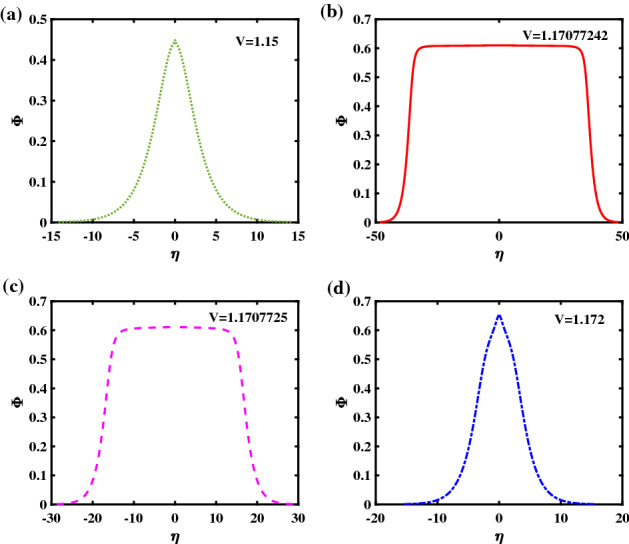
The electrostatic potential profiles corresponding to the Sagdeev pseudopotential curves shown in Fig. 1 are depicted: (**a**) a regular SW (RSW) for $V = 1.15$; (**b**) a flat-top SW (FTSW) for $V =1.17077242$; (**c**) FTSW for $V=1.1707725$, and (**d**) a supersolitary wave (SSW), for $V = 1.172$. The remaining parameter values are: $\beta =0.06439$, $\zeta =0.0001$, $\delta =0.01$, $\mu =16$, and $Q=1$.

In order to gain some insight in the underlying physics and how the curves depicted in Fig. 1 are associated with various observable waveforms, we have plotted in Fig. 3 the electrostatic (ES) potential (pulse) obtained numerically from each curve. Fig. 3a represents the potential profile corresponds to regular solitary wave (RSW) (curve I), while Fig. 3b,d corresponds to FTSW, and SSW, respectively. In Fig. 3c we have plotted the ES potential profile which corresponds to the solitary wave solution obtained for a value of the velocity *V* ($V = 1.1707725$) only slightly above the value of curve (II) ($= 1.17077242$). Figure. 3a shows the usual bell-shaped potential profile of an electrostatic solitary wave, while Fig. 3d shows an supersolitary wave (SSW), possessing a characteristic distorted bell-shape form. Figure 3b,c show a flat top profile corresponding to flat-top solitary waves (FTSW). Although both potential profiles look similar, they differ in the width of the bell curve: one sees that the width of profile (b) passes from a half width of 85.97 down to 20.53 (in dimensionless space units) only, even though the velocity increment does not exceed $10^{-7}$, i.e., 0.00001%. The flat-top soliton topology is very sensitive to variations in the pulse speed, so that the slightest increment in *V* results in a large variation in the width of the potential profile and subsequent loss of the FTSW character, as the speed *V* increases further. This fact suggests that FTSW will solely exist in a well prescribed narrow range in parameters space. Inversely, in terms of diagnostics, when these are observed, one may be able to deduce the plasma characteristics within some level of accuracy.

**Figure 4:**
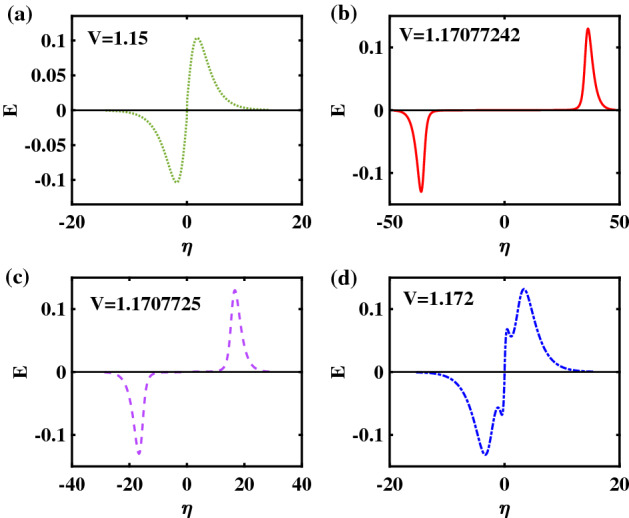
The Electric field profiles corresponding to the Sagdeev pseudopotential curves shown in Fig. 1 (and the ES potential pulses in Fig. 2) are depicted: (**a**) RSW for $V = 1.15$; (**b**) FTSW for $V =1.17077242$; (**c**) FTSW for $V=1.1707725$ and (**d**) SSW for $V = 1.172$. The remaining parameter values are: $\beta =0.06439$, $\zeta =0.0001$, $\delta =0.01$, $\mu =16$, and $Q=1$.

To complement Fig. 3a–d, we have plotted the corresponding electric ($E-$)field profiles in Fig. 4a–d, respectively, which derive from a RSW (Fig. 4a), FTSW (Fig. 4b,c) and SSW (Figs. 4d) configuration, respectively. (Note again that the same color code has been retained as in the previous figure, for quick reference). Note the difference between the bipolar form obtained from a RSW (top left panel) and the wiggly structure that is characteristic of a supersolitary wave (SSW) (bottom right panel)^12^. In the case of the FTSWs depicted in Fig. 4b,c, the distances between the two peaks are relatively large, compared to the characteristic width of the each peak—and also in relation with the standard bipolar forms that dominate space and laboratory observations. However, there is a significant difference between the two, in that the monopolar pulses are farther apart for smaller *V*. This reflects the topology of the Sagdeev pseudopotential in the vicinity of the root.

**Figure 5:**
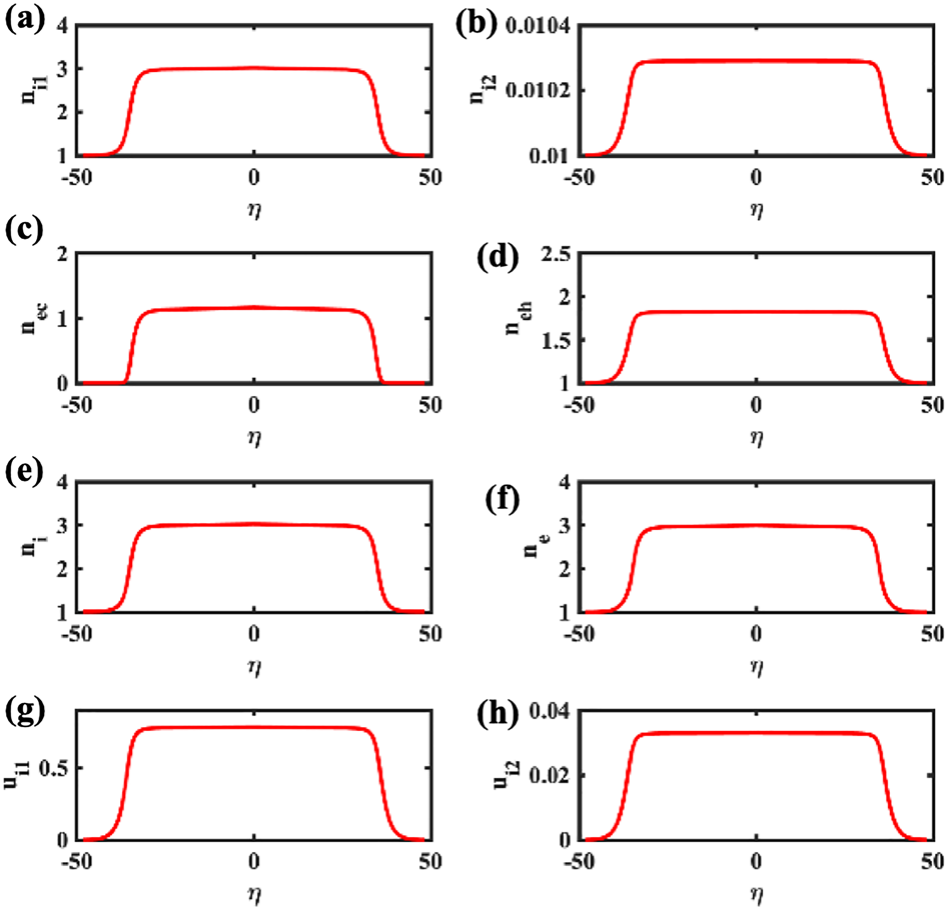
The space profile of the plasma state variables (density of all plasma components, fluid speed for the ion species at the bottom row) are depicted, corresponding to the FTSW profile shown in Fig. 1 (see Curve II), for $V=1.17077242$. The remaining parameter values are: $\beta =0.06439$, $\zeta =0.0001$, $\delta =0.01$, $\mu =16$, and $Q=1$.

In order to understand how the state variables vary in the case of the flat-top (FTSW) solution, in Fig. 5a–h we have plotted the variation of $n_{i1}$, $n_{i2}$, $n_{ec}$, $n_{eh}$, $n_{i}$ (total), $n_{e}$ (total), $u_{i1}$, and $u_{i2}$, respectively, for the value of $V=1.17077242$ corresponding to curve (II) in Fig. 1. All of the curves present a similar flat-top profile that can be henceforth thought of as characteristic of this particular type of electrostatic excitation.

### Significance of the electron parameters

As shown by Ghosh and Iyengar^25^, the coexistence of two electron populations (say, ‘cold’ and ‘hot’ electrons) and the associated electron parameters, such as the relative concentration $\zeta$ and temperature (ratio) $\beta$, play a decisive role in the existence of supersolitary waves. We have adopted a similar rationale and have investigated the dependence of the occurrence and characteristics of FTSW on the cold electron density and temperature.

**Figure 6:**
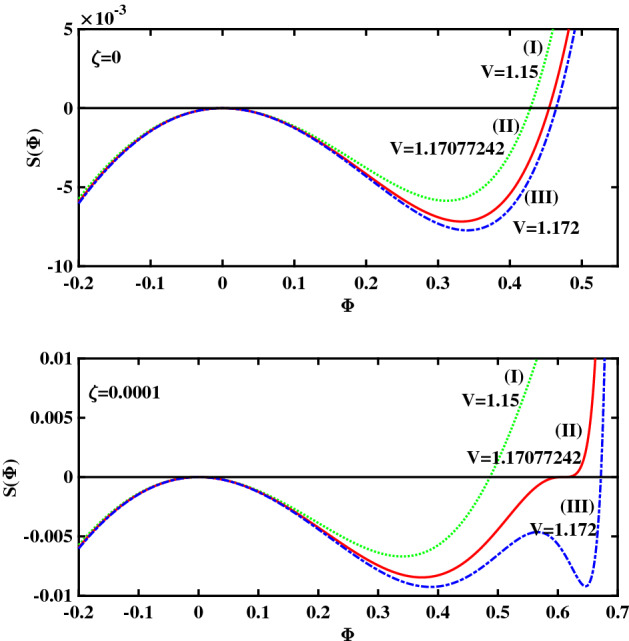
(**a**) Top panel: Sagdeev pseudopotential profiles for different *V* values corresponding to $\zeta =0$ (i.e. no cold electron population); the depicted curves represent regular SWs, for all values of *V*: Curve I for $V = 1.15$; Curve II for $V =1.17077242$ and Curve III for $V = 1.172$. (**b**) Bottom panel: Sagdeev pseudopotential profiles for different V values corresponding to $\zeta =0.0001$ (i.e. a small portion of cold electrons; the depicted curves represent (I) RSW for $V = 1.15$; (II) FTSW, for $V =1.17077242$ and (III) SSW for $V = 1.172$, The remaining parameter values are: $\beta =0.06439$, $\delta =0.01$, $\mu =16$, and $Q=1$.

To illustrate the significance of (the value of) $\zeta$ on various types of nonlinear structures, we have plotted in Fig. 6 the Sagdeev pseudopotential profile obtained for $\zeta =0$ (no ‘cold’ electrons) and for $\zeta =0.0001$ (i.e., just 0.01 % cold electrons added to the mixture), for direct comparison. Upon comparing the two panels in Fig. 6, we see that the simple fact of adding a minute concentration of cold electrons—and keeping all plasma parameters constant—leads to significant modification of the morphology of the entire solitary wave solution, even for the same value of the pulse speed (*V*). It is obvious for the indicative values of *V* considered in Fig. 6 that the plasma only sustains ordinary solitary waves for $\zeta =0$, whereas for $\zeta =0.0001$ one obtains FTSW, and SSW for $V \approx 1.1708$ and $V = 1.172$, respectively.

**Figure 7:**
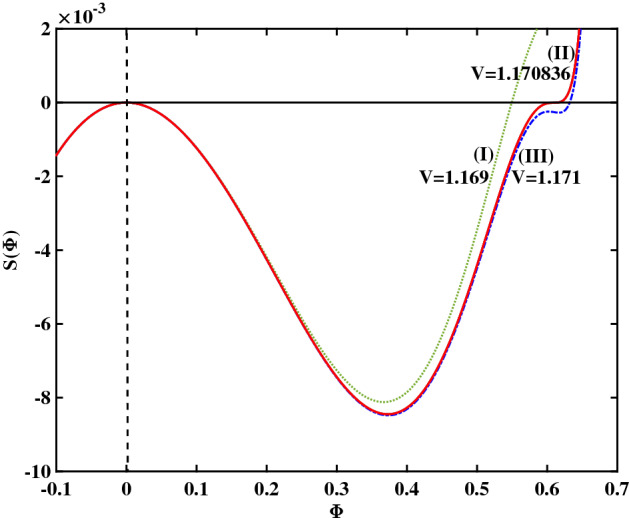
Sagdeev pseudopotential profiles for different V values; the depicted curves represent (I) RSW (in green color), for $V = 1.169$, (II) FTSW (in red color), for $V =1.170836$, and (III) SSW (in blue color), for $V = 1.171$. The remaining parameter values are: $\beta =0.0644$, $\zeta =0.0001$, $\delta =0.01$, $\mu =16$, and $Q=1$.

We have considered different values of the electron temperature ratio ($\beta$), in an attempt to find out if the above trend (i.e., transition RSW–FTSW–SSW upon increasing *V*) is general or, say, if it happened coincidentally. In Fig. 7, we have plotted the Sagdeev pseudopotential curves obtained for $\beta =0.0644$, for different values of *V* (keeping all other plasma parameters constant, as in Fig. 1). In Fig. 7, the three curves (I, II and III) correspond to $V=1.169$, 1.170836 and 1.171, respectively. We notice that this succession of *V* values leads to regular pulses (RSW), flat-top pulses (FTSW) and supersolitons (SSW), respectively.

**Figure 8:**
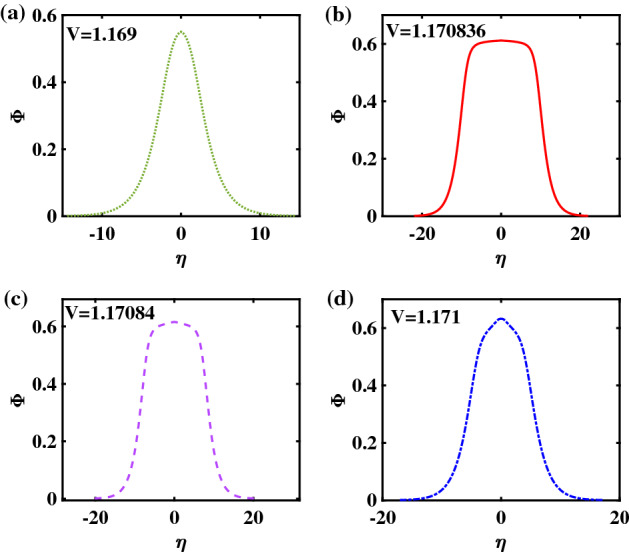
Potential profiles corresponding to the Sagdeev pseudopotential curves shown in Fig. 7: (**a**) RSW, for $V = 1.169$, (**b**) FTSW, for $V =1.170836$, (**c**) FTSW for $V=1.17084$, and (**d**) SSW, for $V = 1.171$. The remaining parameter values are: $\beta =0.0644$, $\zeta =0.0001$, $\delta =0.01$, $\mu =16$, and $Q=1$.

To acquire a better understanding of these structures, the corresponding electrostatic potential profile has been plotted in Fig. 8, for the same parameter values (and keeping the same color code) as in the curves appearing in the previous figure. The top left panel—Fig. 8a—represents a regular bell-shaped pulse (RSW), while Fig. 8b,c show a flat-top structure (FTSW). Unlike in Fig. 2 earlier, no major difference is witnessed in the pulse’s geometrical features (e.g. half-width or height), comparing these two values, although the basic trend is still valid, as above: the faster pulse in Fig. 8c is still slightly narrower and taller though, as expected). Finally, the bottom right panel, i.e., Fig. 8d shows a slightly distorted pulse, characteristic of a supersolitary wave (SSW).

**Figure 9:**
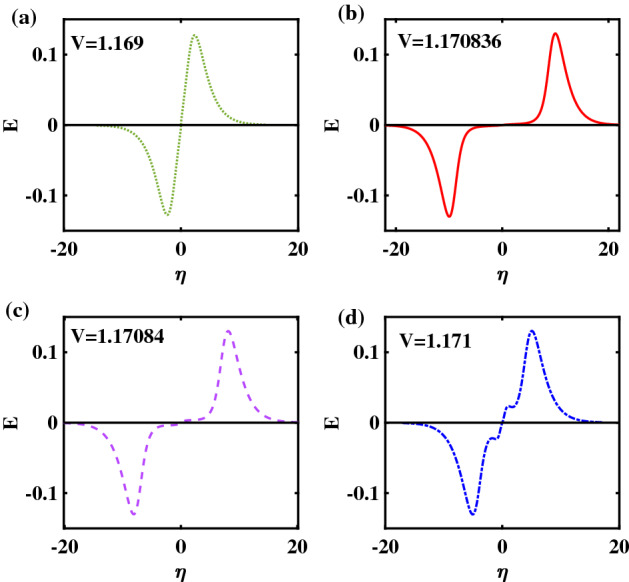
Electric field profiles corresponding to the electrostatic potential profiles plotted in Fig. 8, (**a**) RSW, for $V = 1.169$, (**b**) FTSW, for $V =1.170836$, (**c**) FTSW for $V=1.17084$and (**d**) SSW, for $V = 1.171$. The remaining parameter values are: $\beta =0.0644$, $\zeta =0.0001$, $\delta =0.01$, $\mu =16$, and $Q=1$.

To complement Fig. 8, we have plotted the corresponding E-field profiles in Fig. 9 (for the four panels, respectively). Comparing panels Fig. 9b,c, we note that the flat-top profiles present a finite slope between the two lobes of the electric field. This case is distinct from a pair of monopoles with opposite polarities, that was the case in Fig. 4b.

Comparing the ES potential and electric field profiles for $\beta =0.06439$ and $\beta =0.0644$, we draw the conclusion that a variation in $\beta$ even as small as of the order of $10^{-4}$, the variation in the dynamics of corresponding solitary solutions are quite significant.

**Figure 10:**
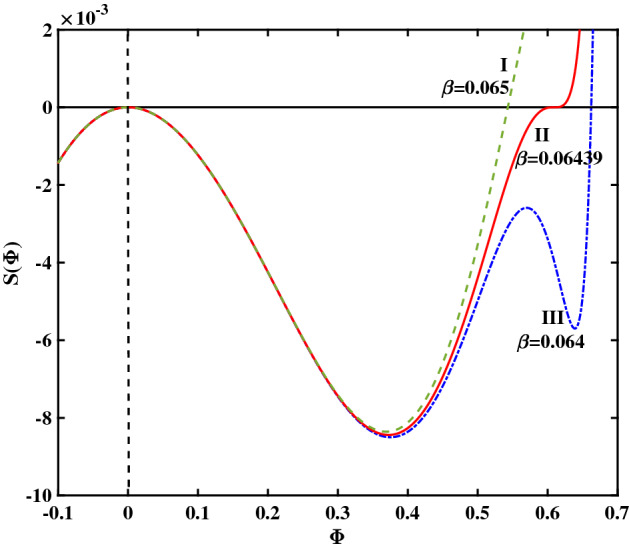
Sagdeev pseudopotential profile transition corresponding to three different $\beta$ values for a fixed *V* value ($V=1.17077242$). RSW (in green color) for $\beta =0.065$; FTSW (in red color) for $\beta =0.06439$, and SSW (in blue color) for $\beta =0.064$. The remaining parameter values are: $\zeta =0.0001$, $\delta =0.01$, $\mu =16$ and $Q=1$.

**Figure 11:**
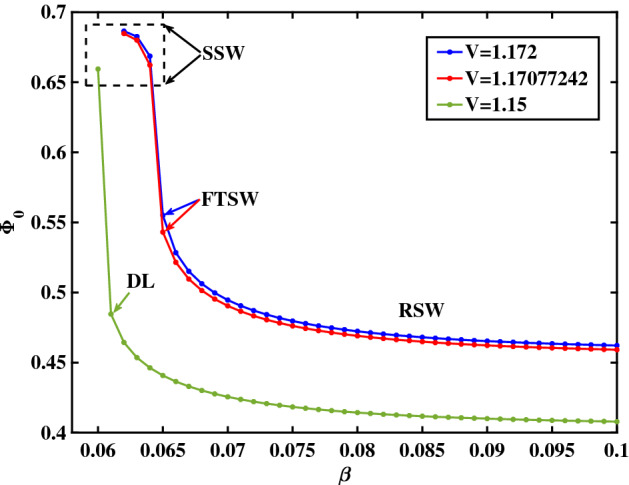
Variation of amplitude $(\Phi _0)$ Vs $\beta$ for three different values of *V*. Blue curve corresponds to $V=1.172$, red curve corresponds to $V=1.17077242$, and green curve corresponds to $V=1.15$. The remaining parameter values are: $\zeta =0.0001$, $\delta =0.01$, $\mu =16$ and $Q=1$.

To gain insight on the influence of $\beta$ on the morphological transition witnessed e.g. in Fig. 10 (upon increasing *V*), we have evaluated the solitary wave characteristics for various values of $\beta$ (keeping the value of *V* fixed). In Fig. 10, curves I, II and III correspond to a regular solitary wave (RSW), a flat-top SW (FTSW) and to a supersolitary waves (SSW), respectively. From the analysis we can infer that, for a given pulse speed (*V* value), upon varying $\beta$ one may obtain a similar looking morphological transition to that shown in Figs. 1 and 7. In Fig. 10, we see that as $\beta$ increases, the SSW transforms to an RSWs; we also infer that, as $\beta$ increases, the amplitude of the solitary wave (pulse) decreases. (In other words, recalling that $\beta ={T_{e, c}}/{T_{e, h}}$, we deduce that the larger the temperature disparity between the two electron populations, the larger the electrostatic potential pulse amplitude will be.) To improve our understanding regarding the amplitude variation, in Fig. 11 we have plotted the amplitude $(\Phi _0)$ variation with respect to $\beta$, by adopting the same (three) values of *V* as in Fig. 6. From Fig. 11, we infer that lower values of $\beta$ result in larger amplitude solitary wave structures, whereas for sufficiently large values of $\beta$, only ”conventional" solitary waves (bipolar E-fields) occur. Once a particular threshold in the value of $\beta$ is exceeded, the amplitude jumps abruptly to a higher value, triggering the formation of SSWs (supersolitons). Either a double layer (DL) or a flat-top structure (FTSW) may lie on the boundary between regular solitary waves and SSWs. One draws the conclusion that a higher value of $\beta$ (i.e. when the "cool" electrons are not "cool enough") rules out the occurrence of ”non-standard" solitary wave profiles.

**Figure 12:**
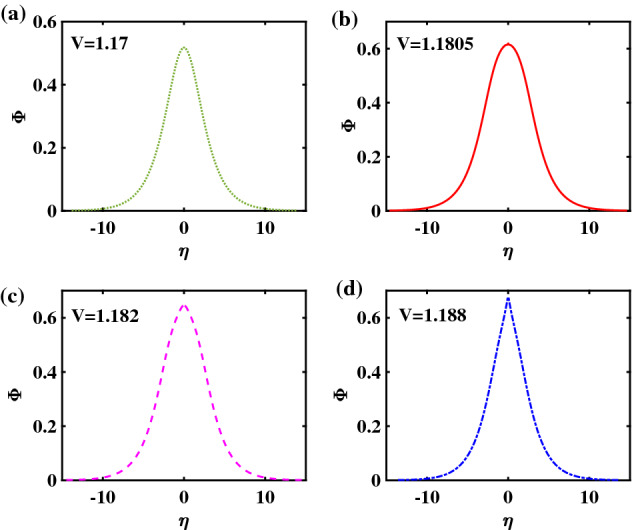
Potential profiles corresponding to $\beta =0.066$. Panels (**a**)–(**c**) represent regular solitary waves (RSWs) for $V = 1.170$, $V =1.1805$ and $V=1.1820$ respectively; panel (**d**) represents a supersolitary wave (SSW), for $V = 1.188$. The remaining parameter values are: $\zeta =0.0001$, $\delta =0.01$, $\mu =16$ and $Q=1$.

To complete the discussion, in Fig. 12 we have plotted the potential profile corresponding to a slightly larger value of $\beta$ ($=0.066$). We see that, as $\beta$ increases beyond a certain value, the occurrence of FTSW is ruled out. It turns out, after a meticulous study of the pseudopotential topology (the relevant plots have been omitted here, for brevity), that the transition from a standard solitary wave to a SSW, upon increasing *V* that is, for large $\beta$, does not involve a FTSW profile. (In other words, the root remains a single root as a pair of new extrema starts developing, resulting in the occurrence of supersolitons.)

**Figure 13:**
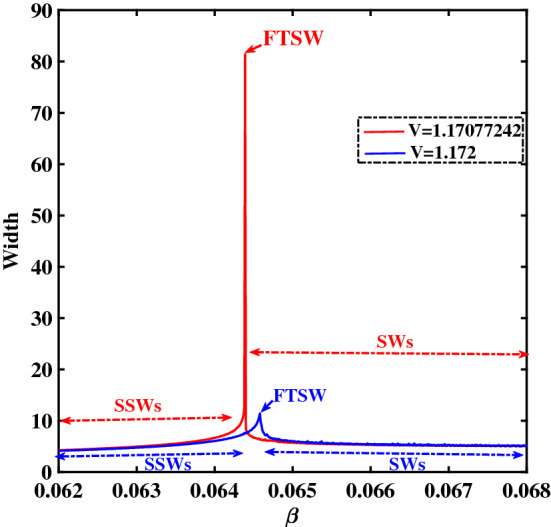
Variation of the width versus the $\beta$ for two different values of *V*. The red curve corresponds to $V=1.17077242$ and the blue curve corresponds to $V=1.172$. The remaining parameter values are: $\zeta =0.0001$, $\delta =0.01$, $\mu =16$ and $Q=1$.

As we mentioned earlier, the electron temperature (ratio) plays a significant role in the morphological transition among different types of solitary structures, which in turn is reflected in the pulse profile width. In order to draw some conclusions or to identify a general quantitative trend regarding the pulse width, in Fig. 13 we have plotted the variation of width with respect to $\beta$ for two different values of *V*. In Fig. 13, the red line corresponds to the width-$\beta$ variation for $V=1.17077242$, whereas the blue line (with lower peak) corresponds to $V=1.172$. We see that, for low $\beta$ values, the width increases with *V*. After reaching a certain value, the width starts decreasing with $\beta$. The peak denotes the position where FTSW occur. The regime on the left hand side of the peak denotes the existence of SSWs, whereas the right hand side denotes the existence of SWs.

**Figure 14:**
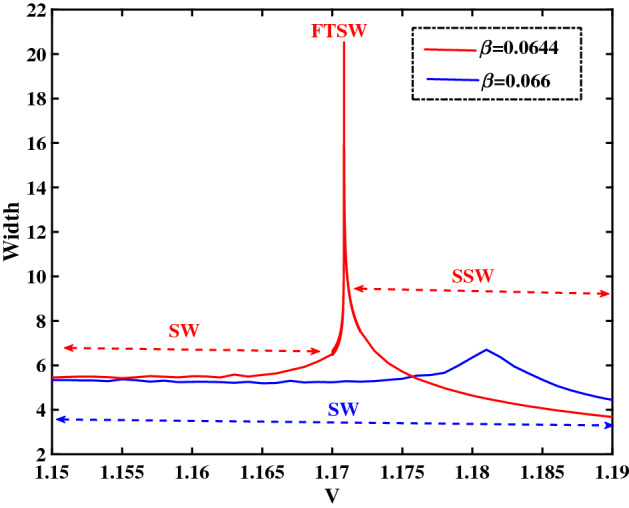
Variation of the width versus the velocity *V* for two different values of $\beta$. The blue curve corresponds to $\beta =0.066$ (as in Fig. 12) and the red curve corresponds to $\beta =0.0644$ (cf. Fig. 8). The remaining parameter values are: $\zeta =0.0001$, $\delta =0.01$, $\mu =16$ and $Q=1$.

In a way analogous to Fig. 13, in Fig. 14 we have plotted the variation of width with respect to the pulse speed *V*, for two different values of $\beta$. We see that, for low *V* values the width increases with *V*; on the contrary, after a threshold is exceeded, the width starts decreasing with *V*. For $\beta =0.0644$ (corresponding to the potential profiles depicted in Fig. 8), we notice a sharp peak (delimiting the region where FTSW will occur). No such peak occurs for $\beta =0.066$, hence no SSWs occur for that value; cf. the potential profiles represented in Fig. 12 for that value of $\beta$.

In earlier work by Steffy and Ghosh^35^, FTSW structures were obtained by considering the combined role of the ion temperature along with the electron temperature. However, from the present analysis, it is clearly understood that the electron temperature plays the crucial role in the dynamics of such extra-nonlinear coherent structures; on the other hand, the ion temperature may alter the dynamics and topology of these structures, but will otherwise not play a decisive role in determining the existence of such structures. A thorough analysis of the role of the ion temperature is currently underway and the results will be reported in a forthcoming article.

## Comparison with observational data

The signature of stretched bipolar pulses or ofbp has been observed in various Earth’s magnetospheric regions, such as the downward current regions of the auroral zone^46,47^, the day side Polar Cap Boundary Layer (PCBL)^2^ and the magnetic reconnection diffusion region^48^ by different satellite missions, e.g. POLAR, FAST, and GEOTAIL. Our theoretical analysis presented above has indeed led to prediction of such "stretched bipolar pulses" (Fig. 9). In this regard, in order to validate our results with real space plasma observations, we have compared our theoretical estimations with observed data referring to stretched bipolar pulses, as reported by Mc Fadden et al.,^4^ in the auroral region.

Based on observational data, the plasma parameters in the auroral region may be obtained as follows: the electron temperature ranges from 0.5 to 5 eV, while the electron density ranges from 5 to 10 cm$^{-3}$^1^. In our model we have chosen: $T_{ec}=0.5$ eV and $n_0=$ 10 cm$^{-3}$, in order to compute all relevant plasma scales, viz., the characteristic length $\lambda _* = 6.54$ m, the light ion acoustic speed $C_{i1}=27.1$ km/s and the proton plasma frequency $\omega _{pi1}=4.14$ kHz; the obtained values are in agreement with satellite observations in the auroral region^1^. For a cold electron concentration $\zeta =0.1 \times 10^{-3}$, and a cold-to-hot electron temperature ratio $\beta =0.0644$, we have obtained ion acoustic flat-top solitary wave solutions whose E-field profile represents an ofbp, moving at a normalized speed $V=1.170836$. To compare our results with the observed solitary waveforms in the auroral region, we have rescaled them, i.e. we transformed our results to the corresponding non-normalized values by using the aforementioned plasma parameters. The analytical computation of the estimation wave parameters are: peak to peak E-field amplitude ($E_{pp})\approx 220$ mV/m, Velocity $(v)=31.6$ km/s, and time duration ($\Delta t) \approx 9.94$ ms. From the observed data of ofbp reported by Mc Fadden et al.,^4^, the peak-to-peak amplitude for the E-field pulse is $E_{pp} \approx 300 mV/m$, while the time duration is of the order of a few millisecond (*ms*). This value closely matches our analytical estimation of the amplitude of the ofbp pulse in the auroral region, as presented above. We see that, by using a simple plasma model, we have predicted the characteristics—and also the conditions for existence—of a FTSW to be compatible with the slowly moving ofbp seen in the Earth’s auroral area.

## Conclusions

Relying on a simple multicomponent (two-ion and two-electron-) fluid plasma model, we have investigated the significance of plasma parameters on the existence of flat-top solitary waves and supersolitons, in contrast with “traditional" pulse-shaped electrostatic solitary waves. FTSW feature a characteristics flat-top (sometimes also called “table top") potential profile. Apart from their unique morphology, FTSW appear to arise at the boundary between two distinct regimes, relating to regular solitary waves and to supersolitons, respectively. It is established by now that a secondary electron component is necessary to sustain an ion acoustic double-layer^25,49^ and may also sustain a super-solitary wave^15^. Similarly, for FTSW also, a marginal composition of cold electron appears to be necessary (and also sufficient) for their existence (at a given window of velocity values). In the context of the pseudopotential framework adopted in our study, the existence domain of different types of nonlinear structures that may occur highly depends on the velocity of the pulse (assuming a certain plasma configuration with fixed parameter values, that is). We have shown that, if certain conditions are met in a given multicomponent plasma configuration, fluctuations in the charge separation (i.e. essentially random fluctuations of the particle density of any of the plasma components, affecting the right hand side of Poisson’s equation (Eq. 14), and thus the pseudopotential $S(\Phi , V)$ topology) may result in the generation of flat-top or elongated pulses or (independently) supersolitary waves (supersolitons). However, this won’t be true for all values of the relevant plasma parameters. Non-standard pulse profiles (amd, in particular, flat-top pulses) therefore exist in a limited region in parameter space, which may explain their rare their appearance in observations.

It may be added, for rigor, that the methodology adopted here provides static predictions of stationary-profile localized traveling modes (solitary waves) but is unable to yield conclusions on the pulse’s dynamical stability. Investigating the pulse stability would require numerical or alternative advanced analytical methods. This is beyond the scope of the present work, and is left for a forthcoming study.

Apart from the concentration of cold electrons, the electron temperature ratio also plays a vital role in determining the topology of FTSW. The existence domain of FTSW is actually very sensitive to the electron temperature ratio $\beta$ (value). Our results are relevant in the interpretation of the signatures of non-conventional electrostatic bipolar pulses, such as stretched or asymmetric bipolar pulses observed in auroral plasma^4^.

Overall, while supersolitary waves may exist in a wide range of parameter values (as confirmed by earlier theoretical studies), it appears that flat-top solitary waves will only occur in a narrow window in the parameter region, which may explain their scarce (but non-negligible) frequency of observation.

Our model has been applied to the auroral plasma, where an electron coexistence (with different temperatures) is typically observed, in effect confirming that the conditions for the occurrence of flat-top or super-solitary waves are indeed met in that environment. Our work should provide a new way of understanding the non conventional localized pulses recorded during satellite expeditions which are known to be crucial in identifying the microphysics across the Earth’s magnetospheric boundary layers.

As discussed above, we were interested in exploring the very basic mechanism(s) contributing to the formation of such “exotic" solitary wave structures. It appears that the electron concentration (coexistence of different populations at different density and temperature) plays a decisive role in the existence of flat-top solitary waves or supersolitonic structures, while the ion characteristics will only affect the features of the observed structures. Generalization to a more realistic model, including e.g. suprathermal particles or an ion beam, will be reported later.

## Data Availability

The data underlying this article will be shared on reasonable request to the corresponding author.
